# INTERPROFESSIONAL EDUCATION (IPE) EVENT DEVELOPS COLLABORATIVE COMPETENCIES IN HEALTHCARE STUDENTS AT MICHIGAN STATE UNIVERSITY

**DOI:** 10.51894/001c.142066

**Published:** 2024-08-30

**Authors:** Aastha Bahl, Carolina Restini, Lauren Azevedo, Jackeline Iseler, Stephanie M. Jalaba, Scott Carlson, Gregory J. Spray, Fabrice Mowbray, Kirsten Waarala

**Affiliations:** 1 College of Osteopathic Medicine Michigan State University; 2 College of Osteopathic Medicine Michigan State University

## INTRODUCTION/BACKGROUND

The Interprofessional Education Collaborative (IPEC) was created in 2009 to advance interprofessional learning experiences for healthcare professional students. Interprofessional competencies support efforts to engage healthcare professional students in interactive sessions on Values and Ethics, Roles and Responsibilities, Communication, and Teams and Teamwork. Despite growing recognition of the importance of interprofessional education (IPE), many osteopathic medical schools still lack formalized IPE in their pre-clerkship curriculum. As healthcare is team-based and osteopathic clinicians must be well-versed in interpersonal collaboration, osteopathic medical students (OMS), as well as other healthcare professional students, should gain exposure to IPE early in their training. Since 2018, Michigan State University College of Osteopathic Medicine (MSUCOM) has hosted four IPE events per academic year, addressing the lack of formal IPE in the pre-clerkship curriculum among OMS and other healthcare professional students.

## OBJECTIVES

The study aims to evaluate the impact of IPE on the Roles and Responsibilities of healthcare professional students’ interprofessional competencies.

## METHODS

A prospective cohort study involved students participating in a virtual IPE event on Roles and Responsibilities. Anonymous, electronically validated questionnaires assessed changes across 16 interprofessional competencies before and after the event (IRB STUDY00007933). Codes were applied for paired analysis. Descriptive statistics used measures of frequency and central tendency. Pre- and post-event scores were evaluated with Wilcoxon signed-rank test. The difference in characteristics (i.e., gender, prior experience with IPE, clinical experience) and competency scores were evaluated with Fisher’s exact and Chi-square tests.

## RESULTS

242 students from Osteopathic Medicine (56.8%), Nursing (22.8%), Communicative Sciences & Disorders (13.5%), and Physician Assistant (6.9%) programs attended the Roles and Responsibilities IPE event, of which 48 surveys were paired (19.8%). Scores across 15/16 (93.8%) competencies increased significantly (P<0.05) from pre- to post-event. Of the 15 competencies, 34.8% showed increased scores, and 50% demonstrated an increase across five or more competencies. Scores were independent of gender, education, or prior experience with healthcare or IPE sessions.

## DISCUSSION/CONCLUSIONS

The Roles and Responsibilities IPE session positively affected interprofessional competencies. The measured interprofessional competencies increased despite high pre-event scores. The increase was not affected by factors influencing success in IPE (i.e., gender, education, and prior experience with healthcare or IPE sessions). The demonstrated effectiveness of IPE events in developing interprofessional competencies reveals a positive assessment of formal IPE in healthcare professional curricula.

